# Investment in HIV/AIDS programs: Does it help strengthen health systems in developing countries?

**DOI:** 10.1186/1744-8603-4-8

**Published:** 2008-09-16

**Authors:** Dongbao Yu, Yves Souteyrand, Mazuwa A Banda, Joan Kaufman, Joseph H Perriëns

**Affiliations:** 1 HIV Department, World Health Organization, Avenue Appia, 1211 Geneva, Switzerland; 2 AIDS Public Policy Project, John F. Kennedy School of Government, Harvard University, & Schneider Institutes for Health Policy, Heller School for Social Policy and Management, Brandeis University, 415 South Street MS 035, Waltham, MA 02454, USA

## Abstract

**Background:**

There is increasing debate about whether the scaled-up investment in HIV/AIDS programs is strengthening or weakening the fragile health systems of many developing countries. This article examines and assesses the evidence and proposes ways forward.

**Discussion:**

Considerably increased resources have been brought into countries for HIV/AIDS programs by major Global Health Initiatives. Among the positive impacts are the increased awareness of and priority given to public health by governments. In addition, services to people living with HIV/AIDS have rapidly expanded. In many countries infrastructure and laboratories have been strengthened, and in some, primary health care services have been improved. The effect of AIDS on the health work force has been lessened by the provision of antiretroviral treatment to HIV-infected health care workers, by training, and, to an extent, by task-shifting. However, there are reports of concerns, too – among them, a temporal association between increasing AIDS funding and stagnant reproductive health funding, and accusations that scarce personnel are siphoned off from other health care services by offers of better-paying jobs in HIV/AIDS programs. Unfortunately, there is limited hard evidence of these health system impacts.

Because service delivery for AIDS has not yet reached a level that could conceivably be considered "as close to Universal Access as possible," countries and development partners must maintain the momentum of investment in HIV/AIDS programs. At the same time, it should be recognized that global action for health is even more underfunded than is the response to the HIV epidemic. The real issue is therefore not whether to fund AIDS or health systems, but how to increase funding for both.

**Summary:**

The evidence is mixed – mostly positive but some negative – as to the impact on health systems of the scaled-up responses to HIV/AIDS driven primarily by global health partnerships. Current scaled-up responses to HIV/AIDS must be maintained and strengthened. Instead of endless debate about the comparative advantages of vertical and horizontal approaches, partners should focus on the best ways for investments in response to HIV to also broadly strengthen the primary health care systems.

## Background

In the past several years, countries have geared up their response to HIV/AIDS, supported by global health initiatives/partnerships (GHIs) such as the Global Fund to Fight AIDS, Tuberculosis and Malaria (GFATM); the United States President Emergency Plan for AIDS Relief (PEPFAR); the World Bank Multi-Country AIDS Program (MAP); and bilateral donors [1]. Others – such as the World Health Organization (WHO), cosponsors of the Joint United Nations Program on HIV/AIDS (UNAIDS), private foundations such as the Gates and Clinton Foundations, and nongovernmental organizations such as Médecins Sans Frontières (MSF, Doctors without Borders) – provide additional support.

These contributions have led to unprecedented attention for AIDS as a health issue, and have enabled many people living with HIV/AIDS (PLWHA) to survive and sustain their families. However, the introduction of antiretroviral (ARV) treatment, along with other interventions that the health sector has made, also exposed the effects of decades of neglect of the health sector, economic crises, structural adjustments, declining public expenditures, and decentralized financing, particularly in Sub-Saharan Africa [2]. This is one reason for the recent revival of the long-standing debate on whether scaling up the responses to specific health problems in developing countries, especially HIV/AIDS, is strengthening or weakening their stretched and fragmented health systems [3-8].

In this paper we examine the case for and against funding AIDS as a specific health issue, and suggest ways to increase the potential for HIV/AIDS funding and programs to deliver further on the promise of health systems development. We first examine the effects of HIV/AIDS itself on health systems, and then enumerate the effects of HIV/AIDS programs on health-system building blocks, by searching and reviewing the available literatures published and available in the public domain using PubMed, POPLINE^®^, AEGiS (AIDS Education Global Information System), Google Scholar™ and other databases. We use the 2007 WHO definition of a health system as "all organizations, people and actions whose *primary intent *is to promote, restore or maintain health," and also use WHO's description of six health-system building blocks: (1) effective, safe, and high-quality health services, (2) a responsive health work force, (3) a well-functioning health information system, (4) equitable access to essential medical products, vaccines, and technologies, (5) a good health-financing system, and (6) strong leadership and governance [9].

## Results

### The effect of HIV/AIDS itself on health systems

In the face of increased pressure caused by untreated HIV/AIDS sufferers seeking health care, resulting in opportunistic infections, it was obvious that in many places health systems were increasingly unable to provide even the most basic levels of preventive and curative care: infant and maternal mortality increased, and decades of public health gains were lost in countries with a high burden of HIV/AIDS [10].

HIV/AIDS increases the demand for health services, and at the same time it reduces the ability of the health service to supply them. Prior to the advent of ARV therapy, half of all medical hospital beds in Sub-Saharan Africa were occupied by people with AIDS. In some countries of East Africa, this proportion reached 80 percent [11]. The effect was that patients with HIV infection crowded out people with other conditions [12,13]. The presence of a large number of AIDS patients with very poor outlooks also contributed to the health professions losing their attractiveness because of increased workloads, exposure to HIV infection, and the work stress that resulted from it [12].

In addition, the ability of the health service to cope with this increased demand declined, because of HIV-related morbidity and mortality and burnout among health professionals. For example, five-to-sixfold increases in health worker illness and death rates were reported for Malawi, and the number of deaths of nurses there represented 40 percent of the average annual output of nurses from training [14].

### The impact of HIV/AIDS programs on health systems

Access to antiretroviral treatment (ART) and other HIV-related services in the health sector likely has both positive and negative effects on the supply of and demand for health services. On the one hand, it is known from industrialized countries and Brazil that patient demand for hospitalization and diagnostic evaluation for opportunistic diseases decreased following the introduction of ART [15,16]. In those settings, this also resulted in savings in health sector expenditures. On the other hand, where health care is less sophisticated and less costly, this is might be offset by the need to provide long-term outpatient care to more and more people requiring lifelong treatment and laboratory tests to monitor ART [17], and scaled-up some HIV prevention interventions, such as male circumcision and prevention of mother-to-child transmission (PMTCT). To date, despite claims that investing in HIV decreases the ability of the health system to produce other health outcomes, the evidence that this is actually happening is largely anecdotal and equivocal [3,7], with as many pointers confirming as arguing against this stance [18,19].

#### Has health service delivery been expanded?

In all countries, HIV/AIDS programs have dramatically improved the delivery of prevention and care services to people living with HIV/AIDS (PLWHA). Although the scaling up of HIV services has likely not escaped the bias of health systems in general to better serve urban and more affluent groups, considerable efforts have been made to overcome equity concerns and reach the most vulnerable, marginalized groups, such as injecting drug users (IDUs), sex workers, and men who have sex with men (MSM) [20-24].

The most spectacular result of WHO's "3 by 5" initiative was to demonstrate that delivering ART through a public health approach is feasible even where health systems are weak overall [25]. Worldwide, around 3 million PLWHA are currently on ART [26]. As effective HIV treatment programs are implemented, hospital admissions plummet and hospital beds are freed up, easing the burden on health care staff throughout the system [27-29]. With the success of the public health model of service delivery and the demonstrable adherence of patients on treatment across the world, and especially in Africa, treatment for AIDS is saving and changing lives [30]. In Brazil, where free ARV treatment has been made available through the national health services since 1996, historical evaluation suggests that the country's ART program led to a 40 to 70 percent decrease in mortality, a 60 to 80 percent decrease in morbidity, an 85 percent decrease in hospitalization [31], and savings of US$ 1.2 billion in health care costs [32].

Equally important, HIV/AIDS prevention and treatment programs in some places have helped to reinvigorate efforts to promote primary health care (PHC) by providing services such as childhood vaccinations, family planning, tuberculosis case-finding and treatment, and health promotion services. In rural Haiti, the "four pillars" approach to HIV prevention and care introduced by Partners in Health radically increased overall patient visits at the Las-Cahobas primary health clinic between July 2002 and December 2003, resulting in greatly increased tuberculosis case-finding: within 14 months of initiation, over 200 TB patients were identified and began receiving directly observed therapy (DOT). Prenatal care visits and immunizations saw similar increases over the same period, going from 100 visits per day to over 500 for both services [18,19]. In Zambia, the PMTCT health post funded by the Global Fund is based in the Reproductive Health Division, which is leading the incorporation of PMTCT into routine maternal health services. In Kenya, the PMTCT strategy and its implementation are integrated with existing reproductive services [33]. In Rwanda, basic HIV care has been added into the primary health centers, contributing to increased use of maternal and reproductive health, prenatal, pediatric, and general health care [28].

Basic health infrastructures have also benefited significantly from the scaling up of responses to HIV. The Brazilian AIDS program has established a specific network of units for the provision of care, often by strengthening existing ones with additional resources [34]. In Lusikisiki, a village in South Africa, there have been significant improvements in terms of reliable electricity, water supply, and telephone and fax services for the clinics. Building and renovation have increased the number of clinics with acceptable nursing services and counseling space [35]. In Haiti, Ethiopia, Malawi, and many other countries, programs provide funds for the construction of health posts, renovation of existing facilities at health centers and hospitals, and training of health personnel [20-24]. In Cambodia, various disease-control programs, including HIV/AIDS, TB, and malaria programs, have been integrated to optimize services and outputs at the district hospital level. The construction and rehabilitation of the district hospitals' common laboratories have been supported. Meanwhile, links and referrals among HIV, maternal and child health (MCH), and reproductive health services have been strengthened, with the expectation that this will improve coverage of PMTCT, MCH, and reproductive health in general [36].

In most cases, scaled-up programs for HIV/AIDS have promoted the public-private partnership needed to provide essential services to target populations, which has enhanced the overall service-delivery capacity of the countries' health systems. In Ethiopia, private labs perform CD4 counts and other HIV/AIDS tests under a quota specified by the Ministry of Health (MOH), and are reimbursed for tests conducted [20]. In Malawi, increased resources support a newly mobilized private nonprofit sector to implement HIV/AIDS activities focused on prevention, care, and support [22]. Recently, PEPFAR and Becton, Dickinson and Company (BD) announced their intention to support the improvement of overall laboratory systems and services in African countries severely affected by HIV/AIDS and TB [37]. In Tanzania, Abbott, a multinational pharmaceutical company, has funded a state-of-the-art outpatient treatment center and clinical labs at Muhimbili National Hospital, which each day will benefit up to 1,000 people with HIV/AIDS and also patients with other chronic diseases [38].

However, there is also evidence of possible negative impacts: in Malawi, the availability of antenatal care services and referrals has decreased, most likely due to provider shortages [22]. There are also concerns that family planning and reproductive health services have been increasingly strained in many places by the decreases and shifts in donor funding away from reproductive health and into HIV programs, unless specifically mandated by donors or national health systems as a needed part of HIV care [8].

#### Have health-sector human resources been expanded?

The scaling up of the response to HIV/AIDS has brought considerable pressure and mixed effects to the health work force in most countries. However, HIV/AIDS treatment per se also has direct beneficial effects on the health work force by keeping HIV-infected medical personnel alive to do their jobs. For example, in Malawi, access to ART had saved the lives of at least 250 out of 1,022 health care workers after 12 months of treatment – workers who were continuing to provide much-needed health services [39].

Increased awareness of the severe health-worker shortage that the need to roll out ART and HIV services helped generate has also led to welcome actions to remedy this problem. For example, in Kenya, the government has agreed that the Clinton Foundation, the Global Fund, and PEPFAR will fund the salaries of more than 2,000 additional health workers for a limited period, after which the government will take over [33]. In Zambia, the UK's Department for International Development (DFID) supports the government's retention scheme aimed at ensuring that health workers are paid additional incentives to work in the most remote areas [40]. Many countries with substantial scale-up programs, such as Thailand, Brazil, Ghana, Ethiopia, and Malawi, have started rapidly training community-level health workers while also gradually expanding the production of higher-level professionals. Malawi has taken a wider approach, focusing on 11 priority cadres because of the extreme nature of its crisis [41]. In Ethiopia, the government decided to hire an additional 30,000 health extension workers in order to place two each in every rural village; 16,000 have already been trained and are providing preventive services and basic curative care at health posts close to their communities [28,40]. The Ethiopian government is also rapidly training and adding nurses and doctors to its health work force. And in Benin, scaling up the HIV/AIDS program has led to recruitment of a large body of non-public-sector professionals into the public sector, which has boosted personnel motivation by providing training, supplies, and equipment [21]. Both the morale and the skills of health workers have been enhanced by means of the training and the incentives such as salary top-ups associated with delivering HIV/AIDS-related services in many areas [22].

Innovative models have been created to meet the health worker shortages resulting from the labor-intensive delivery requirements of HIV services. WHO, together with PEPFAR and UNAIDS, recently developed global recommendations and guidelines on task-shifting [42]. In a recent WHO survey, of 73 low- and middle-income countries, 28 reported having a policy on task-shifting to allow reorganization of tasks among health care workers and the hiring of nonprofessional workers [26]. Research indicates that implementation of task-shifting can reduce the demand for doctor time by 76 percent. Time freed up can be used by doctors to manage complex cases, improve the quality of care, and deliver primary health care [43]. In Malawi, paramedical officers have been trained to provide ARV delivery, with impressive results. More than 81,000 people started ARV treatment through the public sector in Malawi, with only 9 percent of those who begin treatment failing to return and continue uptake of the ART services offered [7]. In Haiti, community health workers are mobilized as the cornerstones of the program providing medical therapy and emotional support to people living with HIV, and also provide much-needed education on HIV prevention and health care to the community [44].

However, scaling up the response to HIV/AIDS can tempt health care workers to take better-paying jobs providing HIV care, and prompt a disproportionate number to work in clinical care and laboratories compared to areas like pharmaceutical support and health education. In Zambia, there are anecdotal reports of localized brain-drains of public-sector health professionals who have switched to well-funded NGO HIV programs. In Rwanda, doctors in the NGO sector reportedly receive six times the salary of their public-sector counterparts [33]. As a result, doctors and nurses move into AIDS care to receive better compensation [8]. In Ethiopia, the health worker situation worsened due to excessive workloads posed by the HIV programs and the lack of incentive mechanisms for retaining staff [20], until salaries were increased recently.

#### Has the health information system been strengthened?

There is a common need to strengthen the generation and use of the information/data required to manage services and to produce and account for results. Evidence is limited on the effects of HIV/AIDS programs on the overall health information system. More and more countries have been reporting on progress toward the Declaration of Commitment that was unanimously adopted in the 2001 UN General Assembly Special Session (UNGASS) on HIV and AIDS: 103 out of 189 countries in 2003; 115 out of 189 countries in 2005; and 147 out of 192 countries as of March 2008 [45]. In Malawi, an electronic patient-monitoring system has been established to replace the manual paper-based system, improving the information management capacity of staff [46]. Sharing of information among different stakeholders has been observed in Benin [21]. In some countries, information sharing among government and civil society organizations has increased, and health information is more available in the public domain [47].

However, it has been reported that countries that have made the effort to implement a single national monitoring system remain burdened by duplicative reporting processes and monitoring missions from multiple programs [47]. Recognizing this, governments and donors are trying to work out strategies for improved coordination of monitoring and information requirements [40]. However, if harmonization is in progress, there is long way to go [47,48].

#### Have procurement and supply management been strengthened?

A functioning procurement and supply management system is necessary to achieve equitable access to essential medicines and technologies. Logistics and supply systems have been improved as a result of investments in HIV/AIDS and other disease-control programs in some countries. In Malawi, national drug procurement now uses the procurement and distribution system from an earlier, parallel procurement system for the disease-control program [22]. In Rwanda and Burkina Faso, HIV drug procurements supported by donor-driven programs have been integrated into the national supply system for essential drugs. In a recent WHO survey, among 66 low- and middle-income countries reporting data on stock-outs of ARV drugs, 41 countries had no ARV drug stock-out in 2007. The remaining 25 countries reported one or more episodes of stock-out of antiretroviral drugs. Globally, 18 percent of all reporting treatment sites experienced at least one stock-out of ARV drugs in 2007 [26], which is much better than the situation of supplying other essential drugs [Perriëns, personal communication].

The establishment of parallel procurement systems for HIV/AIDS programs, similar to ones used to procure other pharmaceuticals and commodities in the public sector, could have negative impact. When such parallel systems bypass government structures and directly interfere with international suppliers, the opportunity to help build the capacity of the country's own procurement and supply management system is missed [21]. In Ethiopia, the MOH outsourced the purchase of drugs and medical supplies from international markets to UNICEF [20]. In many countries, separate supply systems exist for ARV drugs and other commodities funded by the Global Fund and PEPFAR, including those for PMTCT, while drugs for essential obstetric care, contraceptives, and drugs for opportunistic infections and sexually transmitted infections, imported through the government system, are subject to frequent stock-outs [33].

#### Has health financing been improved?

The global scaling up of the response to HIV/AIDS has brought vast resources to bear in the fight against HIV/AIDS. By the end of 2007, AIDS funding was expected to stand at just under US$ 10 billion – an almost fortyfold increase compared to 1996, when it was US$ 260 million [49]. In 2006, it was estimated that US$ 2.5 billion was spent for AIDS by governments using their own public funds. The expenditures by low-income Sub-Saharan African governments for AIDS were estimated between US$ 242.2 million and US$ 390.3 million [50].

While AIDS funding increased, donor support for other public health programs, such as infectious diseases control, has also been increasing in low- and lower-income countries, with one possible exception – population reproductive health, which in absolute constant dollar terms stayed relatively stagnant from 1992 to 2005 (approximately the same amount in 1992, US$ 890 million, as in 2005, US$ 887 million) [51].

In 25 lower-income Sub-Saharan African countries, the domestic public-health spending more than doubled in per capita terms, from US$ 0.31 in 2001 to US$ 0.65 in 2005 [50]. In addition, several GHIs with a focus on AIDS invested a significant amount in health-system-strengthening activities. It is estimated that nearly US$ 640 million of PEPFAR funding was directed towards system-strengthening activities in 2007, including pre-service and in-service training of health workers [28]. Global Fund financing has been used for a wide range of strategies to support health systems, such as salary support and other means of retaining skilled professionals, and it has expanded its support for health system strengthening in the ongoing Global Fund applications [20,52].

However, this picture likely glosses over problems in the allocation of funding for overall health development in developing countries, especially the funding for PHC. For example, total health spending remains critically low in the African region, averaging US$ 32 per capita in 2000. This comprised, on average, US$ 12.5 in government expenditure, US$ 1.2 in donor funds to government, and US$ 16.8 in private expenditure, which included out-of-pocket sources [53,54]. Because few resources have been allocated to PHC, most countries' national health systems are suffering from absolute inadequacy of financial resources [53]. Limited absorptive capacity in some countries is also a concern. Donor funding for HIV/AIDS was comparable to or exceeded the amounts allocated by the national government to the entire health sector in some countries [51]. At the national level, when fiscal ceilings affect the health budget, as in Uganda and Zambia, there is the risk that funds earmarked for HIV and other communicable diseases will crowd out government allocations to priorities such as maternal health, to the payroll, and to infrastructure development for health [33]. Displacement also affects what other donors decide to do with their funds. For example, in Benin, a few partners/donors have canceled or reduced their financial contributions to the subrecipients of GFATM grants because of the Global Fund contributions [21].

At individual level, user fees are the main barriers to adherence to ART [55]. Some informal charges such as transportation and other out-of-pocket expenditures can present a significant barrier to people gaining full access to HIV/AIDS treatment and care services. Quite often, the free ARV package does not cover diagnostics, formal or informal fees, transport to and from the health service, and so forth, which are strong risk factor for mortality [56]

#### Have leadership and governance for health been improved?

Central to all national health systems is the need for effective leadership and governance. The increase in global advocacy for scaling up the response to HIV/AIDS and other major diseases has catalyzed stronger political awareness and leadership for health, in government and in civil society. NGOs and PLWHA are now often included in the decision-making processes through a number of coordinating mechanisms, such as the Country Coordinating Mechanism (CCM) of the Global Fund [47,57]. Together with scaled-up responses, especially in terms of treatment for HIV/AIDS, smarter policies have been initiated that target populations previously neglected in many countries, such as drug users, sex workers, and men who have sex with men (MSM). Planning, transparency of management, monitoring and evaluation, and technical assistance from external sources have been strengthened [47]. AIDS "treatment activism" has promoted access to basic medicines, including ARV drugs for the underserved, and has reduced health care inequities [58].

AIDS activists increasingly advocate for the right of access to universal primary health care. They have also changed the dynamics between health care providers and clients, thus helping prepare health systems for the delivery of chronic care, which requires much more give-and-take between care providers and their clients than does the delivery of acute care [58]. Indeed, it is the activism for AIDS that has created solidarity about health as a concern for humanity, and as part of the evolving paradigm on globalization [59].

In countries like Ethiopia, GHIs supporting the scaling-up programs are in alignment with the national priorities and strategies of the countries [20]. The scaled-up response to HIV/AIDS supported by GHIs has similarly brought changes to policies and strategies even in countries with stronger health systems – for example, by increasing political commitment and by supporting NGO involvement in Central Asian and Eastern European countries and in China, where NGO roles had previously been more politically constrained and limited [23,24]. Kyrgyzstan received and is implementing a GFATM grant for HIV/AIDS services/activities provided primarily by NGOs, focusing on preventive interventions among high-risk groups such as injecting drug users (IDUs), prisoners, sex-workers, and young people. Similar GFATM awards in China have contributed to opening up the political space for NGO participation in the CCM process and for services for marginalized populations such as drug users, sex workers, and MSM [60]. The World Bank programs also supported NGOs to deliver interventions in 2007 in Kyrgizstan [24]. And GFATM grants helped shape the direction of policy by funding HIV harm-reduction efforts for drug users and sex workers in China [47].

However, some observers see PEPFAR's position on abstinence and increased reliance on faith-based agencies as promoting conservative moral and religious views [61,62]. On governance of the health aid structure, there is considerable room to improve the harmonization and coordination among donors and partners at the global, national, district, and facility levels. Uncoordinated proliferation of foreign aid contributes to fragmentation of the health systems of many poor countries [63]. For example, there are at least four committees focused on HIV/AIDS in Tanzania – although there is a clear division of labor [47]. Furthermore, communication between donors and countries is often a one-way street, and the feedback loop from countries is weak. In one survey, 350 stakeholders in 20 countries raised the problem of communication when working with donors [64].

## Discussion

Although accounts of positive and negative effects of AIDS funding are readily asserted [65,66], available evidence on the effects of the scaled-up response to HIV/AIDS on health systems is slim. Many arguments suggesting impacts of HIV investments on health systems are based on anecdotes and speculation, on small pilots, or on early stages of the programs that cannot yet be generalized, and a number of systematic impact studies are still underway. Therefore, it would be imprudent to draw any firm conclusions at this stage.

However, it is likely that global scale-up of responses to HIV/AIDS is having a positive effect on many dimensions of health system performance, especially service delivery and infrastructure upgrading, and that the majority of concerns center on human resources. It is therefore encouraging that major donors and global initiatives are increasingly acknowledging that they must assume responsibility for the health system effects of their actions. Indeed, they are adopting measures to further strengthen health systems while targeting their focused diseases. The World Bank has traditionally focused on strengthening health systems as one of its priorities [63], and now the GFATM is following suit. The GFATM's Sixteenth Board Meeting decided to expand support for health-system-strengthening efforts in coming rounds [28]. PEPFAR is to channel more resources for training and retaining more health workers in the countries hardest hit by HIV/AIDS [29]. And new Global Health Initiatives, such as the International Health Partnerships (IHP), explicitly aim to support building up the health systems of some of the poorest countries [40]. Then, what should we do next?

### Maintain the momentum brought about by investment in HIV/AIDS

It is clear that most countries are far from reaching a level that could conceivably be considered as close to universal access for HIV/AIDS prevention, treatment and care [26]. Donors and country governments should maintain the momentum of the movement that enabled interventions against AIDS to take off in developing countries, and continue to increase investment in HIV/AIDS. The targets of universal access to HIV/AIDS prevention, treatment, and care cannot be reached without increased international investments in many developing countries.

### Maximize the positive synergies of HIV/AIDS programs and health system strengthening

It is time now that we move from the current situation of unplanned "spill-overs" to a more systematic and active management of the synergies between HIV/AIDS programs and health system strengthening in countries [67]. This requires concerted efforts for a policy and technical framework, which will guide actions to avoid threats and maximize the synergies between HIV/AIDS investment and health systems.

### Strengthen HIV/AIDS service delivery and integrate it into the primary health care system

The scaled-up global response to HIV/AIDS began as an emergency response to the crises of high infection and death rates and the urgent need for prevention and treatment efforts. However, in the long run, effective prevention, treatment, and care for HIV/AIDS should be integrated with the existing health service and system because AIDS is a chronic disease. WHO has proposed a public health approach to ART to enable scaled-up access to treatment for HIV-positive people in developing countries, which entails standardized, simplified treatment protocols and decentralized service delivery [68]. Increasingly, the evidence is that this approach works – as long as the health system is strong enough to carry the increased workload of delivering the HIV services. Consequently, we have an historic opportunity to start equipping the primary health care systems in developing countries – which are currently oriented to maternal and child health and the care of acute, episodic illnesses – with the skills to address the chronic health problems that are an emerging threat there. Together with lifelong care for HIV/AIDS, the persisting infectious diseases and emerging noncommunicable diseases in many developing countries mean that their health systems must prepare to become client-perspective-based systems oriented towards both acute illness and chronic care [69-71].

### Advocate for increasing funding for universal primary health care

Primary health care (PHC), as promoted by the Declaration of Alma-Ata thirty years ago, is key to providing good value for money and to enhancing equity of health [70]. There are strong movements to revive and renew PHC as an approach to promote more equitable health and human development [54]. However, the majority of developing countries cannot fund PHC with domestic resources alone. Development partners should therefore assume more responsibility in supporting countries' PHC, in addition to funding treatment and care for HIV, TB, and malaria. It should be recognized that global action for health is even more underfunded than is the response to the HIV epidemic. As stated by the Director General of WHO, sustained commitment is especially important for a disease like HIV/AIDS, where patient survival depends on lifelong access to drugs, but it is also important for funding broader issues such as health system strengthening [72]. New funds are needed for universal primary health care, and we must stop arguing about the sharing of HIV/AIDS funding. The balance needed could be funded with a modest increase in donor funds and sustained effort in developing countries to meet the Abuja target of 15% of government expenditure on health [73]. Activists and NGOs should advocate for both causes – scaled-up response to HIV and strengthening of PHC.

### Better document the impact on health systems of investment in HIV/AIDS programs

More systematic studies should be undertaken on the health systems of different countries, using agreed-upon frameworks and measurements. With partners in PEPFAR and GFATM, WHO is working on the basic principles and framework to guide the future design and implementation of research into this matter. Global health partners should promote both a rigorous appraisal of experiences and a frank dialogue on what has been shown to work and not work in different settings.

## Summary

Current scaled-up responses to HIV/AIDS must be maintained and strengthened. Instead of endless debate about the comparative advantages of vertical and horizontal approaches, partners should focus on the best ways for investments in response to HIV to also broadly strengthen the health system. The evidence is mixed – mostly positive but some negative – as to the impact on health systems of the scaled-up responses to HIV/AIDS driven primarily by global health partnerships. Efforts by countries and their development partners should continue both (1) to maximize the positive synergies of investment in HIV/AIDS and other priority health programs, and (2) to increase funding for universal primary health care, based on the principles and modalities of the Paris Declaration on AIDS Effectiveness – namely, national ownership, alignment, and harmonization [74,75].

## List of abbreviations

ART: antiretroviral treatment; ARV: antiretroviral; CCM: Country Coordinating Mechanism; DFID: Department for International Development; DOT: directly observed therapy; GFATM: Global Fund to Fight AIDS, Tuberculosis and Malaria; GHI: Global health initiative/partnership; IDU: injecting drug users; IHP: International Health Partnerships; MAP: World Bank Multi-Country AIDS Program; MCH: maternal and child health; MOH: Ministry of Health; MSF: Médecins Sans Frontières; MSM: men who have sex with men; NGO: Non-government Organization; PEPFAR: United States President Emergency Plan for AIDS Relief; PHC: primary health care; PLWHA: People living with HIV/AIDS; PMTCT: prevention of mother-to-child transmission; UNAIDS: Joint United Nations Program on HIV/AIDS; UNGASS: United Nations General Assembly Special Session on HIV and AIDS; UNICEF: United Nations Children's Fund; WHO: World Health Organization

## Competing interests

The authors declare that they have no competing interests.

## Authors' contributions

DY developed the initial draft of this essay and contributed to later editing. YS reviewed and commented on successive drafts of this essay. MAB reviewed and commented on successive drafts of this essay. JK reviewed and commented on the later drafts of this essay. JHP reviewed and commented on the successive drafts of the essay and contributed to the policy analysis. All the authors read and approved the final manuscript.
